# Anticipation of periodic events influences cell motility in amoeba proteus

**DOI:** 10.1038/s41598-026-37298-0

**Published:** 2026-01-29

**Authors:** Stephanie Margarete Mueller, Sven Martin, Markus Morawski, Jens Stieler, Max Holzer, Martin Grunwald

**Affiliations:** 1 Paul Flechsig Institute, Centre of Neuropathology and Brain Research, University of Leipzig, Leipzig, Germany; 2 Haptic Research Lab, Paul Flechsig Institute, Centre of Neuropathology and Brain Research, University of Leipzig, Leipzig, Germany

**Keywords:** Single cells, Cell motility, Actin cytoskeleton, Cytoplasmic streaming, UV light, Aversive stimuli, Biophysics, Ecology, Ecology, Microbiology, Neuroscience

## Abstract

All migrating cells (both single-cell organisms and as part of multicellular organisms) sense environmental conditions and respond to physical and molecular cues by changing the direction and speed of cellular movement. Adaptive behaviors enable motile cells to thrive in dynamic environments. A study with slime mold suggested an ability to anticipate dry and cold periods. However, experimental evidence for anticipation in other single-cell organisms is lacking. Here, we investigated whether *Amoeba proteus* can anticipate unfavourable periodic stimuli. *Amoeba proteus* react to blue light (405 nm) by reducing their streaming speed in response to each stimulation. As expected, after four periodic blue light stimulations *A. proteus* presented spontaneous in-phase reduction in streaming speed at the time point when the next stimulation would have occurred. Our results corroborate the claim that single cells are able to anticipate periodic environmental cues and change their behaviour in anticipation of these cues. These findings may have implications for the interpretation of cellular processes in vitro and in vivo even in complex multicellular systems.

**Supplementary Information:**

The online version contains supplementary material available at 10.1038/s41598-026-37298-0.

## Introduction

Amoeboid movement (through actin-dependent crawling) and flagellar swimming are the two most prevalent forms of cell motility in eukaryotic cells^1^. In vivo, cell migration is vital during embryogenesis, tissue homeostasis, wound healing, and defense against infections^2–4^. Furthermore, it is involved in tumor metastasis^2,5^.

All migrating cells, be they single-cell organisms or part of a multicellular organism, sense and respond to environmental conditions. The physical and molecular cues in the vicinity of the cell influence the direction and speed of cellular movement^3,6,7^.

Over the past 15 years, researchers have revisited the age-old question, whether learning and memory play a role in the reaction patterns of single cells, and if they do, how they might come about in organisms with no nervous system^8,9^. Historical and modern studies show that single cells possess at least fundamental learning abilities, such as habituation and avoidance^9–12^. More recent experimental studies suggest that some cells may even be capable of associative learning in the form of classical conditioning^9,13^. However, learning and reaction patterns can be very specific to the species that is studied, making replication difficult.

Recently, it was shown that the slime mold *Physarum polycephalum* could predict periodic environmental changes and would adapt its growth speed in anticipation of the next unfavorable change in environmental conditions^14^. So far, anticipation has not been investigated systematically in any other species. We were interested to investigate whether another single-cell organism would be able to anticipate other unfavorable stimuli.

*Amoeba proteus’* behavioral characteristics have been studied for over 100 years and its reactions to various experimental conditions, such as electrical current, mechanical shock, or changes in illumination, are well reported^15–18^. *Amoeba proteus* is smaller and moves much faster than *Physarum*, but similarly presents with gelatinous ectoplasm and more viscous endoplasm. However, *Amoeba proteus* does not form veins and does not show rhythmic contractile activity. To best of our knowledge, there have not been any studies on anticipation response in *Amoeba proteus*, yet.

To investigate whether *Amoeba proteus* anticipates periodic events, a stimulus was chosen that would be easy to manipulate and fulfil the requirement of eliciting an observable response in movement speed and cytoplasmic streaming of *A. proteus*.

Engelmann was the first to note that a sudden increase in illumination results in pseudopodial contraction, and cessation of locomotion and cytoplasmic streaming^17,19^. This observation has since been confirmed several times^20–22^. In this context, shorter wavelengths elicit a reaction more reliably than longer wave lengths^22^.

In the present study, we expect that *Amoeba proteus* will react to blue light (405 nm) by reducing its streaming speed and that periodic blue light stimulation will cause spontaneous in-phase reduction in streaming speed at the time point of the next anticipated stimulation.

## Materials and methods

### Setup

The amoebae were cultured in a solution of mineral water (Volvic, Danone Deutschland GmbH) and filtered hey infusion with mixed ciliates for food. Solutions used during the experiments were prepared as follows: hey infusion was filtered through syringe filters with Luer-Lock, Polyethersulfon (PES) 25 mm, pore size 0.45 μm (Berrytec GmbH) and mixed 2:1 with mineral water (Table 1). The experimental solution had a pH-value of 6–7. Only light coloured and vigorously flowing specimen were selected for study^17,23^. Each amoeba was carefully taken from the original culture and rinsed twice in a separate dish filled with experimental solution before they were transferred to the test Petri dish (diameter 300 mm). All Petri dishes were made of glass. The depth of the experimental medium in the test Petri dish was approximately 0.4 cm. After transferral the specimen were left in the dark for 15 to 30 min until they attached to the new surface and recovered their usual form and mode of locomotion^24^. Each amoeba was tested separately. The Petri dishes were boiled, dried and subsequently thoroughly rinsed three times with experimental solution between uses. New experimental solution was used for each cell.

**Table 1:** Mineral content of mineral water used for experimental solution according to analysis from the independent SGS Institute Fresenius, Germany.

Minerals	mg/L
Calcium	13.0
Magnesium	9.0
Sodium	12.0
Potassium	7.0
Chloride	16.0
Silicon	31.0
Sulfate	9.0
Bicarbonate	80.0
pH	7
Total mineral content	130 mg/L

The experimental setting and microscope were shielded from external light at all times and were kept in a room with stable temperatures of 21–23 °C. During all experiments, the cells were viewed with a Zeiss Axiovert 10 microscope at 160-fold magnification.

A commercially available USB-microscope camera (MicroOcular 051012-VGA, Bresser GmbH) was used to film the experiment. The experiments were filmed with 30 frames per second. During standard conditions the experimental setting was kept in darkness (infrared light). An infrared lamp cluster of 15 LEDs was used (Kingbright BLO106-15-29 IR emitter 880 nm 40 ° 5 mm, size 26 mm, Kingbright Electronic Europe GmbH) with a peak wavelength of 880 nm. At predefined intervals, the amoebae were exposed to periods of blue light. A lamp cluster of 12 LEDs (TRU Components 5004PCH02 UV-LED 405 nm 5 mm) with a peak wavelength of 405 nm was used for blue light stimulation. The lamp cluster was positioned at 2.5 cm above the Petri dish with the whole petri dish evenly lit. The blue light stimulus had the following parameters at the cell plane: irradiance was 0.28 mW/cm², fluence after 20 s stimulation was 0.005 J/cm². LED heating did not change experimental conditions; no temperature change of the experimental medium in the Petri dish occurred during the experiment.

A priori sample size was calculated using the software G*Power Version 3.9.1.7 (University of Kiel, Germany;^25^. For within-factor comparisons of three repeated measures of streaming velocity (T1, T2, T3) and expected medium to high effect size, with a specified alpha error probability of 5%, the analysis would require a sample size of *N* = 18 to achieve a power of 0.95.

### Experimental procedure

**Fig. 1:**
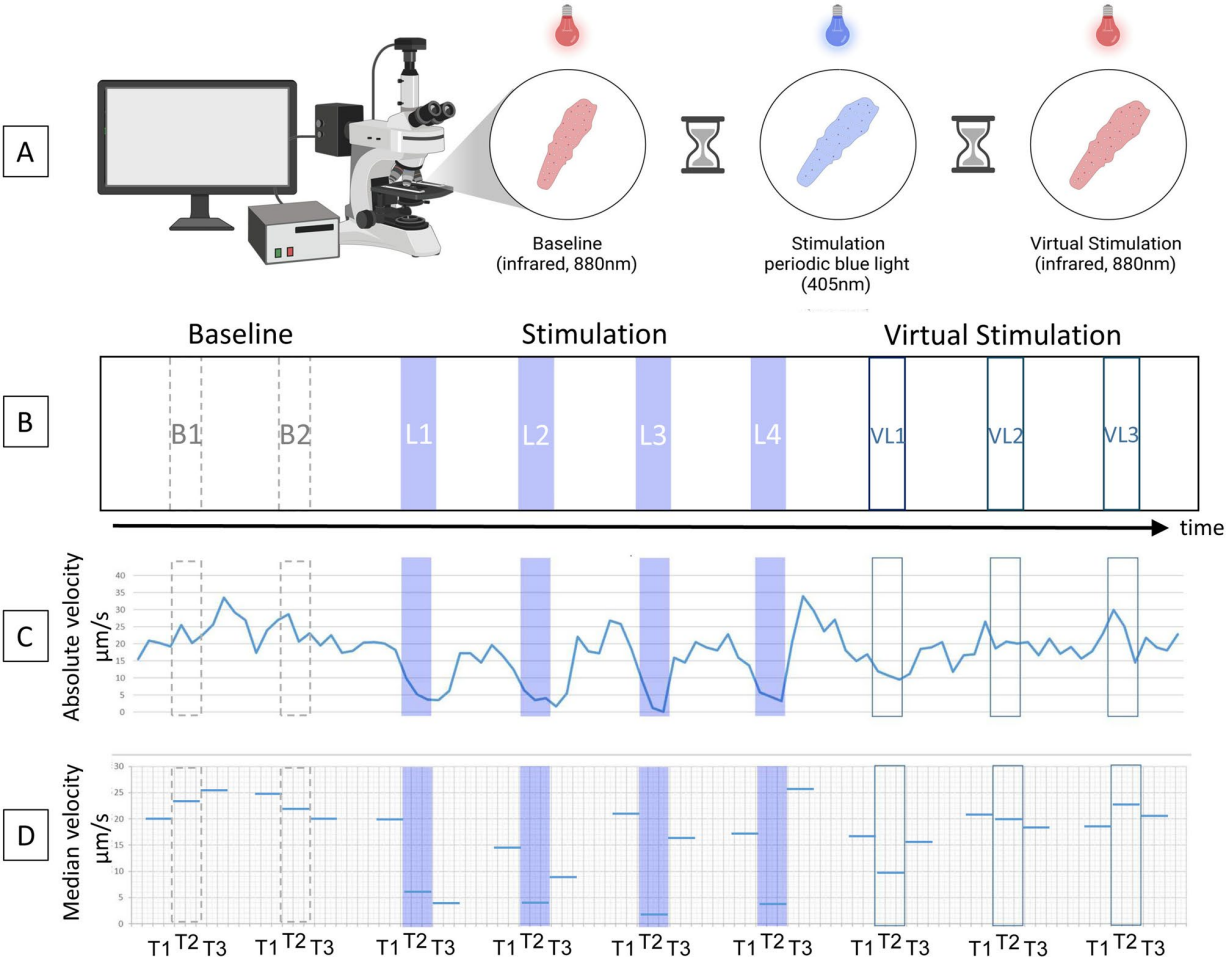
Experimental procedure. **(A)** Schematic representation of the experimental procedure. **(B)** *L1*,* L2*,* L3*, and *L4* indicate the time points of the blue light stimulation. *VL1*,* VL2*, and *VL3* indicate the time points of the virtual stimulations. *B1* and *B2* are reference intervals during baseline. **(C)** Typical time course of cytoplasmic streaming velocities of an *Amoeba proteus* during one experimental session. **(D)** Median streaming velocities calculated for adjacent 20-second intervals before (*T1*), during (*T2*) and after (*T3*) periodic blue light stimulation, virtual stimulation, and reference intervals during baseline. Figure 1A was created in BioRender. Morawski, M. (2026) https://BioRender.com/gyk0gsk.

Each experiment began with a three-minute film sequence of undisturbed amoeboid flow (baseline, B1 and B2, Fig. 1A). The durations of the periodic blue light sequences and the dark periods between blue light exposures were computer controlled. The durations were chosen based on existing literature and pre-studies to prevent fatigue and habituation in the organisms.

Exposure to wavelengths in the range of 330–500 nm have been shown to result in cessation of cytoplasmic streaming after approximately one second of illumination^26^. Similarly, Folger reported average reaction times to increased illumination of 4.66 s^17^. In our experiment, two blue light durations were used: 10 s and 20 s. Longer exposures were not used because the cells began streaming again after approximately 15 s of illumination, which was determined during pre-studies and is in line with previous studies, that have shown, that streaming will begin again after a short time, even if the light is not turned off^21^.

Repeated exposures to light usually result in consistent reaction times, if the dark periods between exposures are long enough. If dark periods are too short, the reactions may become erratic or cease altogether^17^. According to previous studies, darkness should last at least 20 s between repeated exposures to light to secure a reliable reaction and fast reaction time^17^. In our experiment, dark periods were between 60 and 100 s long, to ensure that the amoebae returned to their usual streaming speeds between consecutive exposures.

In short, the specimen received different intervals of blue light (10 vs. 20 s) and different intervals of darkness (60, 70, 80, and 100 s). These irregular intervals were chosen to assess if the cells would show in-phase slow down independently of phase shifted intervals.

After completion of the periodic blue light exposures (see Fig. 1B, L1 through L4), each specimen was filmed for 5 to 7 additional minutes under dark condition. The duration of this film-sequence was computer controlled and resembled three virtual light units (VL1 through VL3) with the same periodic properties as the actual blue light exposures.

Each cell contributed all seven epochs (L1–L4, VL1–VL3) to the analyses plus two epochs baseline (B1 and B2).

### Data Preparation and analyses

**Fig. 2:**
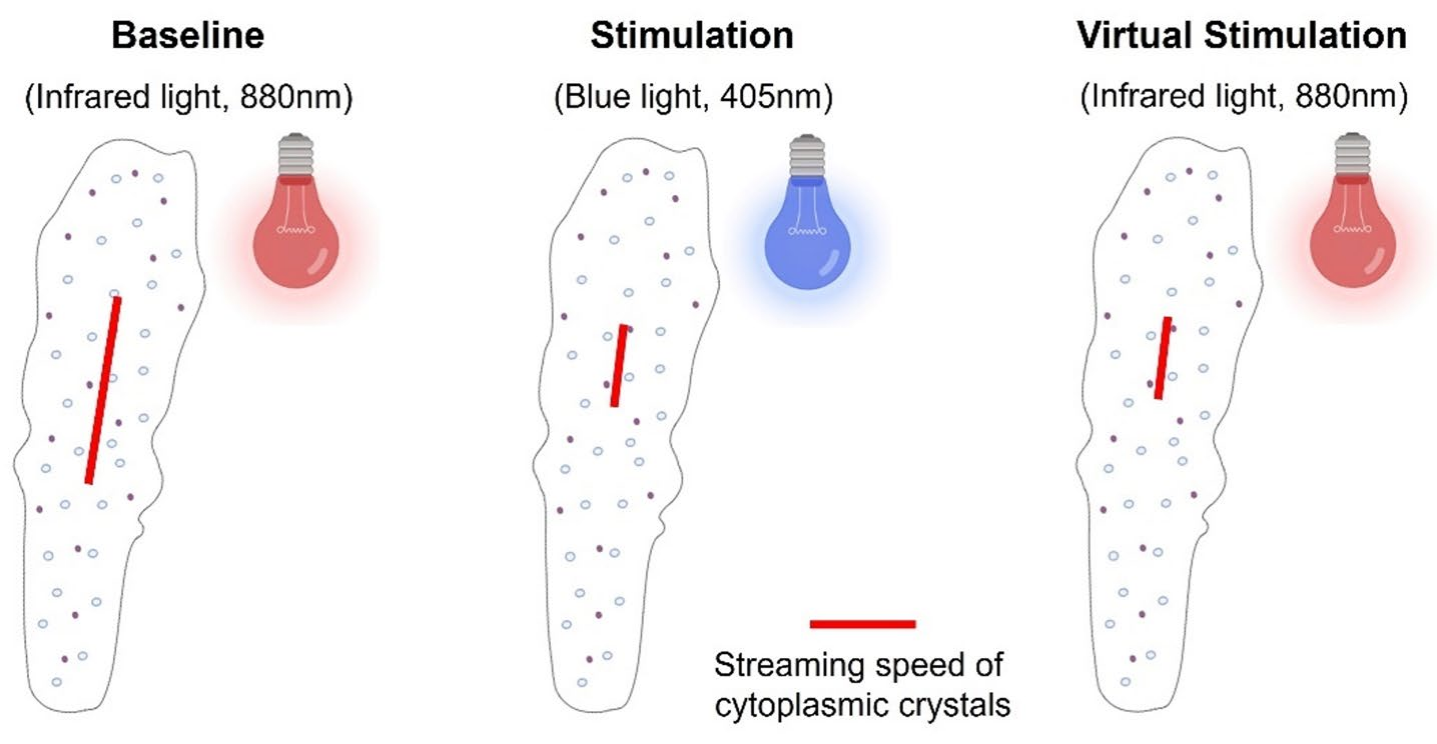
Measurement of streaming velocity of cytoplasmic crystals. Red lines indicate displacement of cytoplasmic crystals during equal time intervals of different experimental parts. Videos were recorded with 30fps. Cytoplasmic streaming was averaged across sequences of ~ 100 consecutive frames. See also Supplementary Figure S1.

The changes of the cytoplasmic crystals served as markers to track the movement of the cells frame-by-frame. Markers were set to the middle of the dominant pseudopod of the amoeba were the most prominent steaming occurred. Streaming velocities were measured across sequences of ~ 100 consecutive frames (equivalent to roughly 3 seconds’ real time; Fig. 2 and Supplementary Figure S1) and saved in micrometer per second (µm/s). The software used to track cytoplasmic streaming was written by author SM and can be accessed with the following link: https://haptiklabor.medizin.uni-leipzig.de/daten. Inter-rater reproducibility was tested on 29 random segments with a length of ~ 100 frames per segment from 5 different cells. Inter-rater correlation (MG and SMM) of streaming speed was high with *r* = .875, *p* < .001. Blinding was not possible in the present setting. Plausibility of streaming speeds was spot-checked using an optical flow tool of the Open-Source Computer Vision Library (OpenCV^27)^.

In the next step, median streaming velocities were calculated during each blue light and each virtual light sequence (T2) as well as adjacent 20-second intervals before (T1) and after (T3) each blue light or virtual light sequence (see Fig. 1C and D). Additionally, median streaming velocities were calculated for six consecutive 20-second intervals during the three-minute baseline sequence. The six intervals during baseline were separated into two groups (baseline1 and baseline2) in order to achieve comparable time-sequences as during blue light and virtual light stimulation. Furthermore, the minimal streaming velocity of each 20-second interval was identified by that 100 frame-sequence (approx. 3 seconds) with the lowest µm/s.

Friedman Tests were performed to compare the median and minimal velocities of three consecutive 20-second intervals (T1, T2, T3) for each of the experimental situations (blue light stimulation, virtual stimulation, and baseline). Alpha was Bonferroni corrected due to multiple Friedman tests with *p*_*crit*_ = 0.05/18 = 0.0028. Post-hoc pairwise comparisons were performed with Mann-Whitney-Tests. IBM SPSS Statistics (Version 29.0.0) was used to analyze the data.

Subgroup analyses of darkness durations and blue light durations were not possible due to small sample size. However, explorative analyses were performed to assess frequency of in-phase reduction of streaming speed. To that end, we divided the minimal velocity during T2 by the median velocity during the preceding dark period (T1). Anticipation was defined as reductions in velocity of more than 20% during virtual stimulation compared to median velocity during the preceding dark period. We chose a 20% difference as cut-off, based on the naturally occurring speed fluctuations during baseline, which were on average 15.7%.

## Results

In our experiment *Amoeba proteus* were periodically exposed to unfavorable blue light (405 nm) stimulations. During standard conditions they were kept in darkness (infrared light, 880 nm). In total 21 cells were tested. Figure 1C shows a typical time course of streaming velocities across the experimental procedure. Median and minimal cytoplasmic streaming velocities are reported for each experimental part in Table 2.

### Statistical analyses

**Fig. 3:**
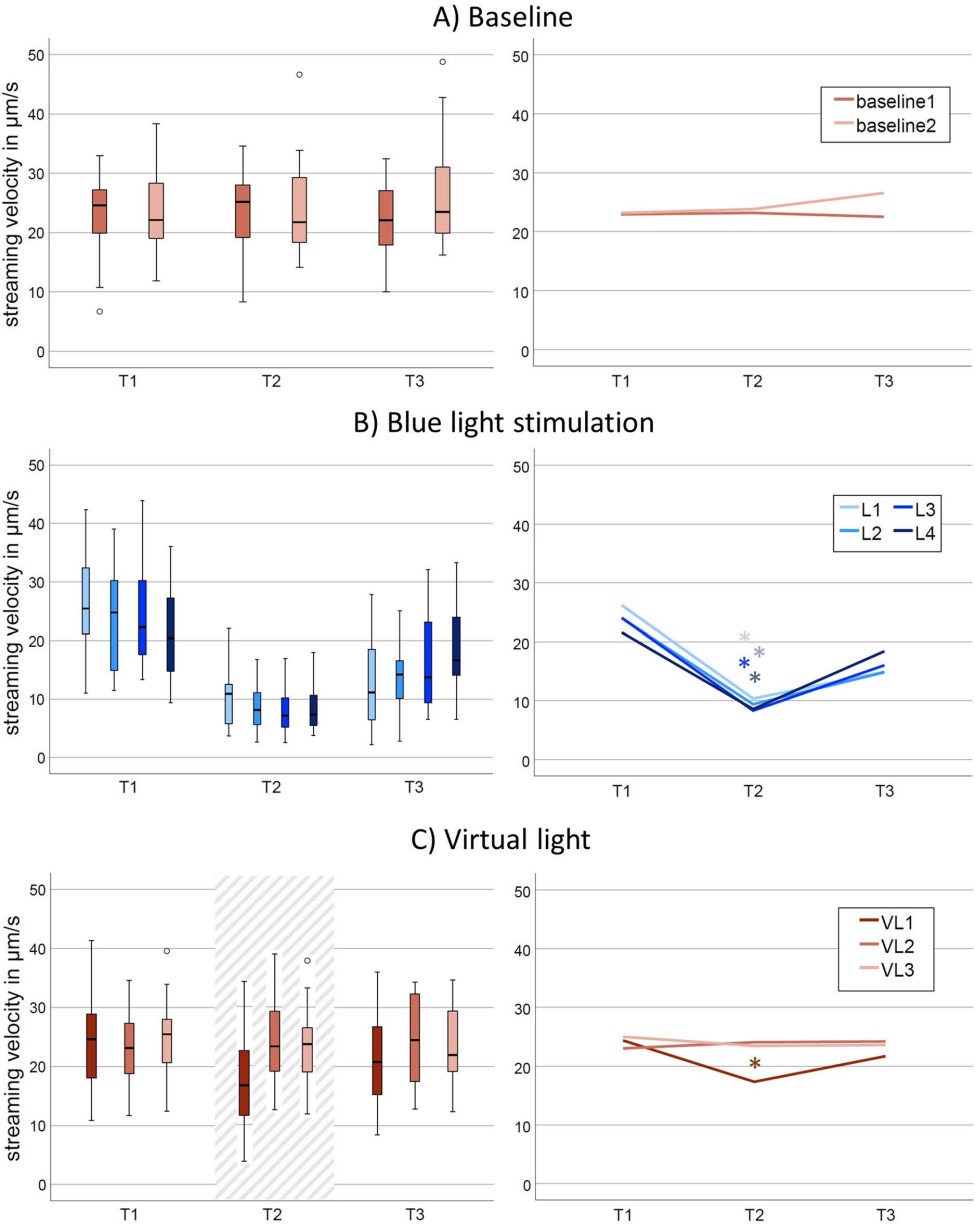
Cytoplasmic streaming velocities of the entire sample during all experimental parts. Streaming velocities were calculated for adjacent 20-second intervals before (*T1*), during (*T2*) and after (*T3*) periodic blue light stimulation, virtual stimulation, and reference intervals during baseline. *L1* through *L4*: blue light stimulation 1 through 4; *VL1*,* VL2*, and *VL3*: virtual light 1 through 3. Left column: Box-plots with 25th and 75th percentiles of each 20-second interval. Small circles: outlier values. The striped area high-lights the three in-phase measurements. Right column: Line-graphs highlight the median streaming velocities of T1, T2, and T3 of each experimental part. Asterisks indicate significant differences between *T1*,* T2*, and *T3* with *p* < .001 (compare Table 2).

As expected, the median streaming velocity of the entire sample was significantly reduced during blue light exposure (T2) compared to before (T1) and after (T3) exposure (L1 through L4; Table 3; Fig. 3). Median streaming velocity 20 s after blue light exposure (T3) was still reduced but returned to baseline velocities before the next blue light stimulation (Supplementary Table S1 for post hoc pairwise comparisons). In accordance with the blue light exposures, the median streaming speeds during T1, T2, and T3 of the virtual light segments were compared. During the virtual light segments, the median streaming velocity was significantly reduced during T2 of the first virtual light (VL1) but not during VL2 or VL3 (Fig. 3C). The median streaming velocity during the time point when the next periodic blue light stimulation would have occurred (VL1T2) was significantly lower than during the 20-second segments before (VL1T1, *z*=-3.39, *p* < .001) and after (VL1T3, *z*=-3.42, *p* < .001) the virtual stimulation. For comparison, we performed exploratory analyses whether cytoplasmic streaming velocity during VL1T2 differed from any of the baseline reference intervals. The median streaming velocity during VL1T2 was significantly lower than the median streaming velocities of all six baseline measurement time points (Chi²=24.39, *p* < .001), while the six baseline measurement time points did not differ from one another (Chi²=5.514, *p* = .356). Median streaming velocities during the second and third virtual light (VL2T2 and VL3T2) did not differ from any of the six baseline measurement time points (Chi²=4.684, *p* = .698) nor from the 20-second segments before and after (VL2T1, VL3T1, VL2T3, VL3T3) virtual stimulation (Chi²=2.114, *p* = .833).

**Table 2:** Median and minimal cytoplasmic streaming velocities and statistical comparisons of the entire sample.

Median streaming velocities (20 s intervals)														
	T1				T2				T3					Effect size
	median	IQR 25–75	95% CI	%Δ (T1 → T2)	median	IQR 25–75	95% CI	%Δ (T2 → T3)	median	IQR 25–75	95% CI	Chi²	*p*	Kendall‘s W
baseline1	24.60	19.49–27.23	20.47–26.57	3.46	25.45	19.45–28.23	21.11–26.96	-6.13	23.89	18.07–27.26	20.34–25.75	0.40	0.819	0.010
baseline2	21.70	17.85–28.34	20.94–27.16	0.28	21.76	17.28–30.07	21.11–28.33	7.95	23.49	19.93–31.30	23.71–31.68	4.67	0.097	0.111
L1	24.53	19.94–32.39	23.41–29.86	-58.79	10.11	5.78–12.53	7.91–13.17	10.29	11.15	5.86–18.48	9.95–19.95	26.57*	< 0.001	0.633
L2	23.66	14.90–30.23	20.94–28.43	-67.58	7.67	4.89–11.08	7.20–11.97	84.88	14.18	9.35–17.34	11.39–18.54	24.38*	< 0.001	0.580
L3	21.54	15.66–30.23	21.08–28.34	-67.60	6.98	4.58–10.21	6.54–10.37	93.98	13.54	8.06–23.13	12.33–19.98	32.67*	< 0.001	0.778
L4	20.41	14.50–27.29	18.83–25.49	-64.04	7.34	5.39–10.70	6.74–10.70	126.57	16.63	14.07–26.14	14.85–22.10	21.89*	< 0.001	0.576
VL1	23.28	18.05–28.90	21.70–28.67	-30.28	16.23	11.56–22.53	14.07–21.74	26.12	20.47	15.21–26.70	18.43–25.62	15.52*	< 0.001	0.370
VL2	22.93	17.86–27.29	20.85–26.41	1.09	23.18	18.34–29.26	21.23–27.71	1.77	23.59	17.48–32.27	21.20–27.80	1.24	0.538	0.029
VL3	25.46	20.17–28.02	22.94–28.50	-6.91	23.70	18.73–26.86	21.06–26.62	-7.30	21.97	18.92–29.85	21.28–26.49	5.70	0.058	0.142
**Minimal streaming velocities (approx. 3 s)**														
	**T1**				**T2**				**T3**					**Effect size**
	median	IQR 25–75	95% CI	%Δ (T1 → T2)	median	IQR 25–75	95% CI	%Δ (T2 → T3)	median	IQR 25–75	95% CI	Chi²	*p*	Kendall‘s W
baseline1	19.51	13.49–23.02	16.29–21.23	7.59	20.99	17.65–24.71	17.56–22.98	-8.62	19.18	14.47–22.45	17.08–21.28	2.10	0.350	0.052
baseline2	18.72	14.82–24.13	16.54–22.73	-3.10	18.14	13.94–25.95	17.34–22.96	9.48	19.86	17.09–25.12	19.80–26.27	3.06	0.217	0.073
L1	19.87	17.02–24.00	18.68–23.81	-81.73	3.63	1.73–5.16	2.74–7.26	7.71	3.91	1.81–10.41	4.47–11.57	26.00*	< 0.001	0.619
L2	19.11	12.99–24.00	17.44–23.09	-81.74	3.49	1.18–3.86	2.34–5.50	50.72	5.26	1.83–10.15	4.80–10.55	30.95*	< 0.001	0.737
L3	17.32	12.92–24.97	17.47–23.16	-89.09	1.89	0.98–4.46	1.87–5.04	194.71	5.57	2.96–11.43	4.94–10.26	34.67*	< 0.001	0.825
L4	17.06	11.83–22.06	13.94–20.68	-83.94	2.74	1.78–3.69	2.26–4.29	162.04	7.18	4.47–12.03	6.28–11.57	24.00*	< 0.001	0.632
VL1	17.49	15.33–22.91	16.70–23.06	-25.96	12.95	7.72–18.56	9.77–17.16	23.94	16.05	11.25–22.24	12.91–19.74	14.86*	< 0.001	0.354
VL2	19.42	14.23–23.95	17.70–22.53	-3.66	18.71	13.93–24.87	16.34–22.44	6.79	19.98	14.78–25.04	15.58–21.87	1.24	0.538	0.029
VL3	20.53	15.93–23.68	18.63–23.09	-7.79	18.93	16.01–21.90	17.17–22.57	-7.87	17.44	14.78–24.52	15.78–22.22	1.90	0.387	0.048

IQR: inter quartile range; %Δ change of median streaming speed in percent; 95% CI: Confidence Interval based on 1000 bootstrap samples. Chi²: Friedman Tests; Asterisk: significant difference with Bonferroni corrected p-value < 0.0028. L1 through L4: blue light stimulation number 1 through 4; VL1, VL2, and VL3: virtual light stimulation 1 through 3. Median streaming velocities during adjacent 20-second intervals before (T1), during (T2), and after (T3) periodic blue light stimulation, virtual stimulation, and baseline. Minimal streaming velocities are indicated by those 100 frames segments (approx. 3 s) with the slowest cytoplasmic streaming of each 20-second interval.

### Explorative analyses

**Fig. 4:**
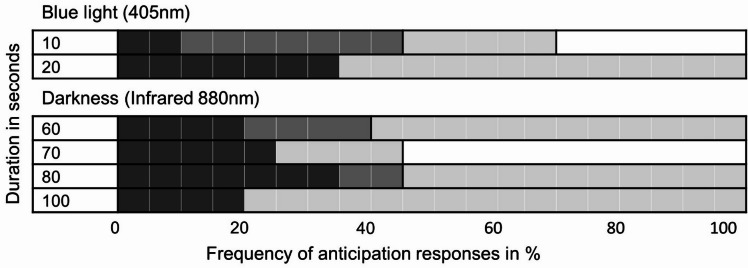
Frequency of anticipation responses. The gray levels of the bars indicate the number of anticipations: black denotes three-times, dark gray denotes two-times, light gray denotes one-time, and white denotes zero anticipation responses. Anticipation was defined as reductions in streaming velocity of more than 20% during virtual light compared to median speed during preceding dark period. Instances when the minimum speed occurred a little earlier or later than the virtual stimulation were not included. Number of repeats: blue light: 7 (10s), 14 (20s); darkness: 5 (60s), 4 (70s), 8 (80s), 4 (100s).

Additionally to the repeated measures analyses, we were interested to explore the frequency of in-phase reductions of streaming velocity across different durations of blue light and darkness. Frequency of decelerations during VL was analyzed descriptively for all individual cells. Decelerations were considered relevant if cytoplasmic streaming slowed by more than 20%. (Please see Table 3 for rate of change in cytoplasmic streaming speed per cell.) Accordingly, deceleration of cytoplasmic streaming occurred in 90.5% of all specimen during the first virtual light. In 38.1% of all specimen, it occurred during the first and second virtual lights, and in 28.6% of the specimen, it occurred during all three virtual stimulations (Fig. 4). Reductions in steaming speed during virtual stimulation occurred with similar frequency independently of the duration of the dark periods and blue light stimulations (see Fig. 4). Two specimen (9.5%) did not show any deceleration during VL. These two cells also had faster absolute streaming speeds during blue light exposures compared to the other cells. However, we could not discern any differences in morphology, rate of change, or baseline streaming speeds of these two cells compared to the other cells that were tested.

**Table 3:** Rate of change in cytoplasmic streaming speed in percent.

	Rate of change in streaming speed in percent								
Amoeba #	B1	B2	L1	L2	L3	L4	VL1	VL2	VL3
**1**	***-28,58***	27,69	-96,07	-92,99	-93,06	-90,98	***-55,39***	***-61,35***	***-36,12***
**2**	-16,05	-18,53	-87,39	-90,08	-95,18	-85,46	***-39,91***	13,96	-17,12
**3**	-2,20	18,12	-67,07	-67,63	-68,00	-48,67	***-37,91***	20,76	***-28,46***
**4**	-12,84	-14,38	-75,62	-80,95	-79,07	-85,46	***-24,62***	6,16	0,00
**5**	***-27,89***	***-23,64***	-95,06	-73,74	-89,77	-80,07	***-26,64***	***-22,12***	***-24,01***
**6**	6,69	-21,29	-81,33	-77,20	-99,44	-81,02	***-47,32***	-9,07	-19,57
**7**	-10,85	***-27,31***	-83,07	-95,36	-84,62	-93,59	***-65,20***	45,78	***-22,71***
**8**	-15,90	-4,35	-74,68	-83,29	-93,86	-85,85	***-94,51***	***-21,14***	-15,95
**9**	-7,91	-10,32	-80,69	-91,86	-85,22	-86,16	***-48,65***	***-50,91***	***-30,78***
**10**	-6,57	-11,23	-86,58	-93,90	-94,57	-90,78	***-46,26***	***-21,20***	-18,52
**11**	12,75	***-61,80***	-90,23	-83,43	-97,83	-88,39	***-60,49***	-8,78	-15,52
**12**	-5,91	***-24,92***	-94,29	-86,96	-89,43	-92,56	***-82,55***	52,13	***-26,94***
**13**	***-20,61***	-3,86	-85,01	-88,06	-97,22	-92,36	***-50,53***	-12,28	***-35,73***
**14**	***-26,13***	-16,38	-91,98	-87,46	-98,36	-71,45	***-94,82***	22,04	-17,73
**15**	-0,21	-19,13	-90,91	-83,51	-88,85	-93,32	***-38,23***	-15,20	-6,96
**16**	-7,22	***-20,04***	-91,86	-93,98	-87,05	-92,38	***-54,94***	***-45,68***	***-20,33***
**17**	-17,84	0,40	-90,15	-95,73	-81,90	-89,68	***-29,58***	***-55,89***	***-39,76***
**18**	***-22,91***	-19,48	-91,86	-93,98	-88,55	-92,67	***-50,30***	***-62,37***	***-22,75***
**19**	-9,63	***-39,09***	-98,39	-97,17	-89,61	-86,44	***-20,87***	2,32	***-23,07***
**20**	-17,71	30,06	-43,58	-72,02	-69,89	-84,91	4,67	-4,61	-17,68
**21**	-18,53	***-22,00***	-39,82	-56,01	-46,51	-61,60	2,92	-11,56	4,58
**Median**	-12,84	-18,53	-87,39	-87,46	-89,43	-86,44	-47,32	-11,56	-21,55

B1 and B2: baseline 1 and 2; L1 through L4: blue light stimulation number 1 through 4; VL1, VL2, and VL3: virtual light stimulation 1 through 3. Rate of change was calculated as follows: ROC=min_velocity_T2/median_velocity_T1*100 – 100. For minimal and median streaming velocities at T1and T2 see Table 2. Bold and italicized numbers indicate reductions in streaming speed of more than 20% during baseline and virtual light; during bluelight stimulation (L1 through L4) all cells showed a decrease in streaming speed of more than 20%.

## Discussion

In the present study, we used an aversive stimulus (blue light with 405 nm) to investigate if *Amoeba proteus* are able to anticipate periodic events. More precisely, we found that *A. proteus* reduce their migration speed in anticipation of the next aversive stimulus.

As expected, all studied cells showed a pronounced reaction to blue light exposure. During blue light exposure, cytoplasmic streaming slowed down in all studied cells and came to a full stop in some (Table 2), which is in line with previous reports^17,26^. Furthermore, streaming began again after a short time while the cells were still exposed to the blue light^17,21^.

For eukaryotic cells without chloroplasts – both single cell organisms and mammalian cells – ultraviolet (UV) light is toxic through DNA lesions^28^. In vitro, blue light and UV exposure of non-lethal intensities affects the directionality and speed of mammalian cell movement^29,30^. Anecdotally, we observed that the direction of streaming changed in 60% of the cells after at least one of the blue light sequences and that many of the cells formed new pseudopods after blue light exposure. Similarly, *Amoeba proteus* have previously been reported to exhibit negative phototaxis when exposed to a lateral light beam^26^.

The median cytoplasmic streaming velocities measured during the present experiment were *Mdn* = 23 μm/s (IQR 17–31 μm/s) during undisturbed streaming, with peak streaming velocities of more than 40 μm/s (Fig. 3). In the present study, cytoplasmic streaming velocity was measured at the center of the cell where the most vigorous streaming occurs^31^. Other authors have investigated the migration speed by measuring the displacement of the whole cell during a given time. Those studies reported that *A. proteus* show an average movement speed of 1.7 to 10 μm per second^32–34^. Therefore, in *A. proteus* cytoplasmic streaming is several times faster than its migration speed.

The central analysis of the present study confirmed that four periodic applications of blue light were sufficient to induce anticipation in nearly all specimen. In-phase deceleration of cytoplasmic streaming occurred in 90% of all cells during the first virtual stimulation. Roughly 30% of all specimen exhibited reduced cytoplasmic streaming during all three virtual stimulations. That means, that a third of the cells retained the periodic rhythm for several minutes after the last stimulation occurred. In all, *A. proteus* showed slightly stronger anticipatory response than *Physarum*^14^: In Physarum, spontaneous slowdown at the time of virtual stimulation occurred in only 35–60% of the samples depending on the duration of the periods between stimulations^14^. Only 10% of the slime molds exhibited three “spontaneous in-phase slowdowns“ [sic], while 40% of the organisms failed to undergo any^14^. In our study, only 9.5% of Amoebas did not show any in-phase slowdowns. However, magnitude and persistence of anticipation are difficult to compare between different studies, because different species, different experimental stimuli, and different biological parameters were used as dependent variables.

In the present study, the duration of the dark periods between blue light stimulations were varied between 60 and 100 s. Shorter dark periods were not used, to ensure that the amoebae returned to their usual streaming speeds between consecutive exposures. Descriptive statistics suggest that the frequency of anticipation responses was similar for all dark periods (Fig. 4). Therefore, in the present study, *Amoeba proteus* showed anticipatory in-phase reduction of velocity independently of the periodic rhythm that was used.

Attempts have been made to explain the mechanisms behind the predictive process. One prevailing theory is a memristive model – an electronic circuit equipped with a memory-resistor (a memristor) – which simulates the prediction of periodic events in complex materials^14,35,36^. However, it is still under debate what the memristive element could be in living cells. In *Physarum*, rhythmic contractile activity of its veins has been shown to be associated with intracellular biochemical oscillations^37,38^. Some authors think that the ability of the slime mold to sense periodic events stems from a range of biochemical oscillators in the cell, which may be able to tune to the periodic structure of environmental changes^12,39,40^. While several such biochemical oscillators have been found in *Physarum polycephalum*^12^, they may not be as obvious in other single-cell organisms. So far intracellular biochemical oscillations have been reported in glycolysis, cAMP signals, and calcium cycles, among others^41–43^.

As a motile cell with actin microfilaments, *A. proteus* depends on the ongoing assembly and disassembly of its actin structures to allow for rapid responses to extracellular cues^44^. It is feasible that the actin system of *A. proteus* may contain oscillatory processes. Oscillating responses of the actin system have been observed in leukocytes^45,46^. More recent studies have investigated the possibility of intrinsic oscillatory processes in the actin system of *Dictyostelium discoideum* and found a resonance peak at a stimulation period of around 20 s^47^. The oscillatory dynamics may be associated with Rac1 activity and other actin-regulatory proteins^48,49^. Future studies should investigate if oscillatory processes of the actin system are the basis for anticipatory responses in *A. proteus*.

### Limitations

Averaging over 20 s bins may obscure finer timing. However, we chose to average across 20 s segments because the longest UV stimulations lasted 20 s and the reaction times of the amoebae varied between stimulations: sometimes a cell would react instantly, sometimes it would take a few seconds. Furthermore, during some UV stimulations the cell would slow down and then speed up again within the 20 s stimulation interval, in other cases it would slow down for more than 20 s. An exemplary full velocity trace of one amoeba is depicted in Fig. 1C, where the differences in reaction patterns between stimulations are visible.

Averaging is a conservative and robust tool to analyze complex data. We have reported the minimal streaming speed (100 frame segment) of each 20 s interval to substantiate the findings. Future studies should try to analyze full velocity traces.

### Outlook and future key experiments

To elucidate the biophysical origin of the observed memory-like and anticipatory behavior, future work will systematically probe the mechanochemical regulation of actin dynamics, contractility, calcium signaling, and energy availability. Sub-disruptive perturbation of actin polymerization using latrunculin B or cytochalasin D will test whether slow actin structural relaxation underlies history dependence, with reductions in hysteresis and memory timescale serving as key readouts. Partial inhibition of myosin II with low-dose blebbistatin will clarify the role of contractility in shaping oscillation amplitude and timing, while assessing whether memory persistence remains unaffected. The contribution of calcium signaling as a fast-upstream trigger will be examined by intracellular Ca²⁺ buffering with BAPTA-AM, linking calcium transients to updates of the internal actin-based state.

Finally, mild ATP depletion using metabolic inhibitors will probe the energetic dependence of the system, testing whether ATP-driven actin dynamics sustain oscillations and memory. Together, these experiments will, in a follow-up study, establish a mechanistically grounded framework connecting actin-based cellular biophysics to emergent memory and anticipatory behavior.

## Conclusion

Anticipation in single-celled organisms is an increasingly recognized phenomenon, reflecting adaptive behaviors that enable these simple life forms to thrive in dynamic environments. This ability to anticipate future events can be viewed as a fundamental characteristic of living systems, extending from unicellular organisms to complex multicellular entities. Moreover, the theoretical underpinnings of anticipation within single-celled organisms hinge on simple systems of pattern recognition^35^. Elementary anticipatory systems, which have been explored in various models, suggest that unicellular organisms can develop anticipatory strategies by learning sequences of environmental inputs^39^. This notion not only supports the concept of anticipatory processing at a cellular level but also suggests that such capabilities may arise through evolutionary selection, directly influencing the survival and reproductive success of these organisms in fluctuating environments^12^. Future studies should investigate how anticipatory processing at the cellular level may influence dynamic processes in multicellular organisms and how these processes could be used in regenerative medicine^50^.

## Supplementary Information

Below is the link to the electronic supplementary material.

Supplementary Material 1

## Data Availability

All data generated or analysed during this study are included in this published article (and its Supplementary Information files).
