# Supplementary material for: Anticipation of periodic events influences cell motility in amoeba proteus
Source: Sci Rep. 2026 Jan 29;16:4762. doi: 10.1038/s41598-026-37298-0 (PMC12873120; doi:10.1038/s41598-026-37298-0)
Supplement: Supplementary file 1 — Supplementary Material 1 [file 41598_2026_37298_MOESM1_ESM.docx]

**Supplementary Table S1.** Post hoc pairwise comparisons (Wilcoxon for dependent samples)

| Pairwise comparisons T1, T2, and T3 | | | | | | | | |
| --- | --- | --- | --- | --- | --- | --- | --- | --- |
|  |  | median | IQR 25-75 |  | median | IQR 25-75 | *z* | *p* |
| Baseline1 | T3 | 23.89 | 18.07 - 27.26 | T2 | 25.45 | 19.45 - 28.23 | -.643 | .520 |
| Baseline2 | T3 | 23.49 | 19.93 - 31.30 | T2 | 21.76 | 17.28 - 30.07 | -1.616 | .106 |
| L1 | T3 | 11.15 | 5.86 - 18.48 | T2 | 10.11 | 5.78 - 12.53 | -2.172 | .030 |
| L2 | T3 | 14.18 | 9.35 - 17.34 | T2 | 7.67 | 4.89 - 11.08 | -3.007 | .003 |
| L3 | T3 | 13.54 | 8.06 - 23.13 | T2 | 6.98 | 4.58 - 10.21 | -3.806 | <.001 |
| L4 | T3 | 16.63 | 14.07 - 26.14 | T2 | 7.34 | 5.39 - 10.70 | -3.622 | <.001 |
| VL1 | T3 | 20.47 | 15.21 - 26.70 | T2 | 16.23 | 11.56 - 22.53 | -3.424 | <.001 |
| VL2 | T3 | 23.59 | 17.48 - 32.27 | T2 | 23.18 | 18.34 - 29.26 | -.434 | .664 |
| VL3 | T3 | 21.97 | 18.92 - 29.85 | T2 | 23.70 | 18.73 - 26.86 | -.411 | .681 |
| Baseline1 | T2 | 25.45 | 19.45 - 28.23 | T1 | 24.60 | 19.49 - 27.23 | -.709 | .478 |
| Baseline2 | T2 | 21.76 | 17.28 - 30.07 | T1 | 21.70 | 17.85 - 28.34 | -.087 | .931 |
| L1 | T2 | 10.11 | 5.78 - 12.53 | T1 | 24.53 | 19.94 - 32.39 | -4.015 | <.001 |
| L2 | T2 | 7.67 | 4.89 - 11.08 | T1 | 23.66 | 14.90 - 30.23 | -3.945 | <.001 |
| L3 | T2 | 6.98 | 4.58 - 10.21 | T1 | 21.54 | 15.66 - 30.23 | -4.015 | <.001 |
| L4 | T2 | 7.34 | 5.39 - 10.70 | T1 | 20.41 | 14.50 - 27.29 | -3.783 | <.001 |
| VL1 | T2 | 16.23 | 11.56 - 22.53 | T1 | 23.28 | 18.05 - 28.90 | -3.389 | <.001 |
| VL2 | T2 | 23.18 | 18.34 - 29.26 | T1 | 22.93 | 17.86 - 27.29 | -.469 | .639 |
| VL3 | T2 | 23.70 | 18.73 - 26.86 | T1 | 25.46 | 20.17 - 28.02 | -2.128 | .033 |
| 20-second interval after stimulation vs. 20-second interval before following stimulation | | | | | | | | |
|  |  | median | IQR 25-75 |  | median | IQR 25-75 | *z* | *p* |
|  | L1 T3 | 11.15 | 5.86 - 18.48 | L2 T1 | 23.66 | 14.90 - 30.23 | -3.146 | .002 |
|  | L2 T3 | 14.18 | 9.35 - 17.34 | L3 T1 | 21.54 | 15.66 - 30.23 | -3.632 | <.001 |
|  | L3 T3 | 13.54 | 8.06 - 23.13 | L4 T1 | 20.41 | 14.50 - 27.29 | -2.535 | .011 |
|  | L4 T3 | 16.63 | 14.07 - 26.14 | VL1T1 | 23.28 | 18.05 - 28.90 | -2.294 | .022 |

**Supplementary Figure S1**

**
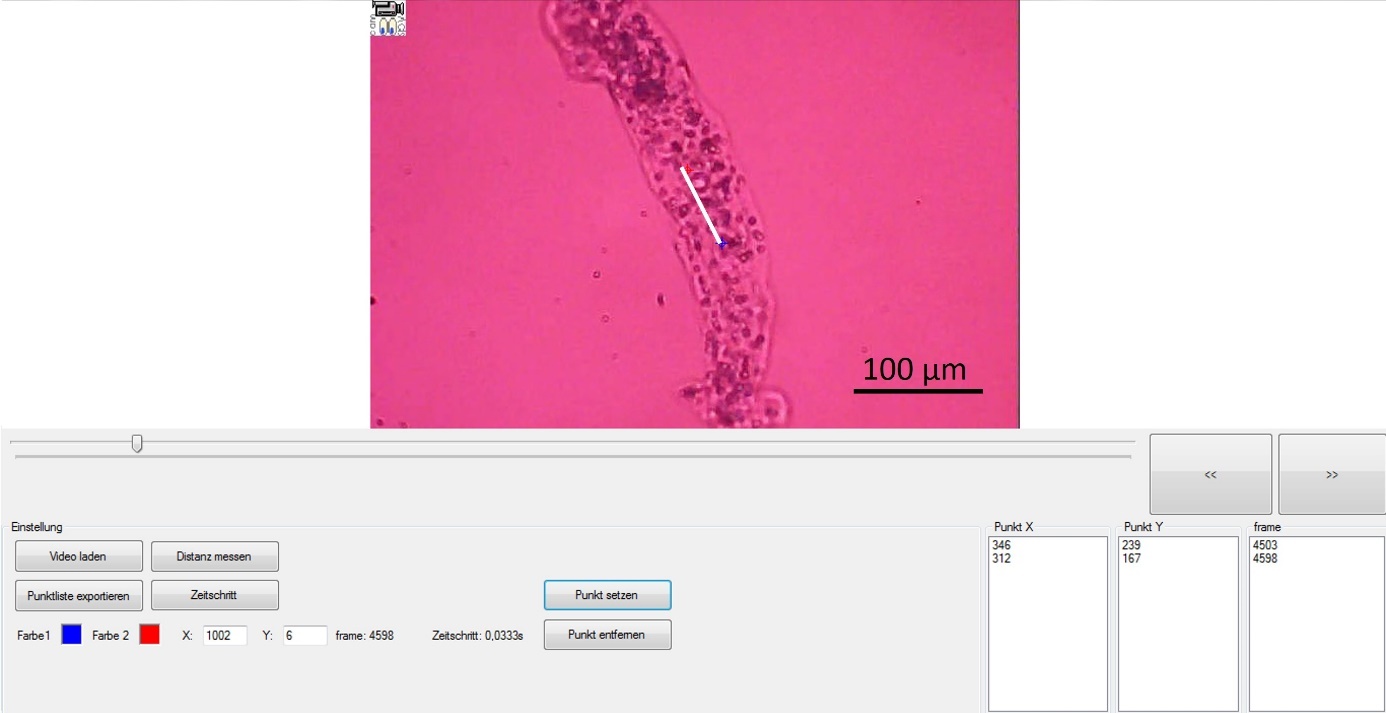
Figure S1. Measurement of streaming velocity of cytoplasmic crystals.** White line indicates displacement of cytoplasmic crystals after roughly 3 seconds (100 frames) of streaming during baseline. Videos were recorded with 30fps. Cytoplasmic streaming was tracked frame by frame. The software used to track cytoplasmic streaming was written by author SM.
